# A Tailored SMS Text Message–Based Intervention to Facilitate Patient Access to Referred Community-Based Social Needs Resources: Protocol for a Pilot Feasibility and Acceptability Study

**DOI:** 10.2196/37316

**Published:** 2022-10-11

**Authors:** Tyler Lian, Hadley Reid, Abigail Rader, Sarah Dewitt-Feldman, Elmira Hezarkhani, Elizabeth Gu, Malik Scott, Kate Kutzer, Sahil Sandhu, Carolyn Crowder, Kristin Ito, Howard Eisenson, Janet Prvu Bettger, Ryan J Shaw, Allison A Lewinski, David Y Ming, Hayden B Bosworth, Leah L Zullig, Bryan C Batch, Connor Drake

**Affiliations:** 1 Department of Population Health Science Duke University School of Medicine Durham, NC United States; 2 School of Medicine Duke University Durham, NC United States; 3 Lincoln Community Health Center Durham, NC United States; 4 Trinity College of Arts & Science Duke University Durham, NC United States; 5 Department of Family Medicine and Community Health Duke University School of Medicine Durham, NC United States; 6 Department of Orthopaedic Surgery Duke University School of Medicine Durham, NC United States; 7 Duke University School of Nursing Durham, NC United States; 8 Center of Innovation to Accelerate Discovery and Practice Transformation (ADAPT) Durham Veterans Affairs Medical Center Durham, NC United States; 9 Department of Medicine Duke University School of Medicine Durham, NC United States; 10 Department of Pediatrics Duke University School of Medicine Durham, NC United States; 11 Department of Medicine Division of Endocrinology Duke University School of Medicine Durham, NC United States

**Keywords:** text messaging, primary health care, social determinants of health, needs assessment, community health centers, vulnerable populations

## Abstract

**Background:**

Health care providers are increasingly screening patients for unmet social needs (eg, food, housing, transportation, and social isolation) and referring patients to relevant community-based resources and social services. Patients’ connection to referred services is often low, however, suggesting the need for additional support to facilitate engagement with resources. SMS text messaging presents an opportunity to address barriers related to contacting resources in an accessible, scalable, and low-cost manner.

**Objective:**

In this multi-methods pilot study, we aim to develop an automated SMS text message–based intervention to promote patient connection to referred social needs resources within 2 weeks of the initial referral and to evaluate its feasibility and patient acceptability. This protocol describes the intervention, conceptual underpinnings, study design, and evaluation plan to provide a detailed illustration of how SMS technology can complement current social needs screening and referral practice patterns without disrupting care.

**Methods:**

For this pilot prospective cohort study, this SMS text message–based intervention augments an existing social needs screening, referral, and navigation program at a federally qualified health center. Patients who received at least one referral for any identified unmet social need are sent 2 rounds of SMS messages over 2 weeks. The first round consists of 5-10 messages that deliver descriptions of and contact information for the referred resources. The second round consists of 2 messages that offer a brief reminder to contact the resources. Participants will evaluate the intervention via a survey and a semistructured interview, informed by an adapted technology acceptance model. Rapid qualitative and thematic analysis will be used to extract themes from the responses. Primary outcomes are implementation feasibility and patient acceptability. Secondary outcomes relate to intervention effectiveness: self-reported attempt to connect and successful connection to referred resources 2 weeks after the initial referral encounter.

**Results:**

The study received regulatory approval in May 2021, and we anticipate enrolling 15-20 participants for this initial pilot.

**Conclusions:**

This protocol presents detailed implementation methods about a novel automated SMS intervention for social care integration within primary care. By sharing the study protocol early, we intend to facilitate the development and adoption of similar tools across different clinical settings, as more health care providers seek to address the unmet social needs of patients. Study findings will provide practical insights into the design and implementation of SMS text message–based interventions to improve social and medical care coordination.

**International Registered Report Identifier (IRRID):**

DERR1-10.2196/37316

## Introduction

### Background

Social determinants of health lead to profound inequities within a wide range of health outcomes [1]. Accumulated research on social determinants of health has resulted in many health care systems implementing interventions in the clinical setting, which screen patients for unmet social needs (eg, food and housing) [2]. Patients with identified needs can then be referred to relevant community resources and services (eg, food pantries and public housing programs). Previous studies demonstrate the potential benefits of social needs screening and referral, including the resolution of identified social needs, improvements in health, and decreases in avoidable health care utilization [3-6].

Patients, however, often face structural and personal barriers in the process of contacting and initiating services [7,8], which threaten the realization of these longer-term benefits. Despite success in the implementation of screening and referral, patients’ actual use of referred services is mixed: the reported proportion of successful connection (ie, initiation of referred services) ranges from 32% to 64% for interventions that screen and refer individuals for multiple social needs in primary care settings [9,10]. Further, clinics often have cost constraints that limit the ability to provide labor-intensive case management services to ensure referral success and service initiation [11]. Therefore, it is critical to design scalable interventions that address barriers and facilitate service access. In particular, patient-reported barriers related to engaging with referred resources suggest that different modes of information delivery may be important to explore [12].

Leveraging SMS text messaging within social needs screening and response interventions could offer an accessible, affordable, and scalable alternative to in-person or telephonic case management and service navigation [13]. According to the Pew Research Center, more than 97% of Americans own a cell phone among different income, education, age, and race and ethnicity groups [14]. The ubiquity of cell phones has been accompanied by a large number of studies that have used SMS text messaging as an intervention medium to promote health self-management and behavior change [15], including weight loss [16,17], chronic care management [18-22], medication adherence [23-25], smoking cessation [26], mental health [27,28], and clinic attendance [29,30]. There is evidence that people living in underresourced communities also benefit from SMS for various health-related applications [31-36]. Thus, SMS may be an effective technology to reach patients who are disproportionately affected by systemic social inequity and, as a result, present with high levels of unmet social need.

Given the broad evidence base for SMS text message–based interventions, integrating automated SMS text messages within social needs screening and response may be an optimal application of SMS text messaging. Previous studies have identified increased intervention dosage (ie, the number of contacts between a patient and the care team) as a significant factor associated with successful social needs resource connection [37], especially within the first 30 days of referral [38]. Increasing in-person or telephone follow-up, however, may be challenging given that screening and referral for social needs is often already a resource-intensive and time-consuming process for providers [39,40]. Further, frequent telephone calls may be costly for low-income patients with limited calling plans [41] and difficult to schedule given patients’ work, caregiving, and other responsibilities.

These considerations motivate the development of an automated and scalable intervention that uses SMS text messages to deliver tailored information and reminders about referred resources. SMS text messaging may be a potential modality to increase patient contact without increasing the workload of busy providers and staff. As an asynchronous mode of communication, SMS text message delivery also allows patients to access messages at their convenience and stores them for easy reference. Although SMS text messages alone cannot address complex, system-level barriers to connection, they may be able to address more proximate barriers such as gaps in the presentation and distribution of referral information, which extend the capacity of the care team to navigate other challenges.

### Study and Protocol Aims

Our multi-methods pilot study aims to assess the feasibility and acceptability of a novel SMS text message–based intervention to promote patients’ connection to referred resources for unmet social needs within 3 weeks of the initial referral. This protocol describes the intervention, development, study design, and evaluation plan for the pilot. Our objective is to provide a detailed illustration of how SMS technology can complement an existing social needs screening, referral, and navigation program at a federally qualified health center (FQHC) without disrupting usual clinical care. This protocol makes a novel contribution by presenting one approach to using automated messages that can readily scale to other clinical settings. Results from the pilot can inform the adaptation of SMS text message technologies for social needs screening and response to increase the uptake of community-based resources and health-related services.

### Conceptual Model and Outcomes

**Figure 1:**
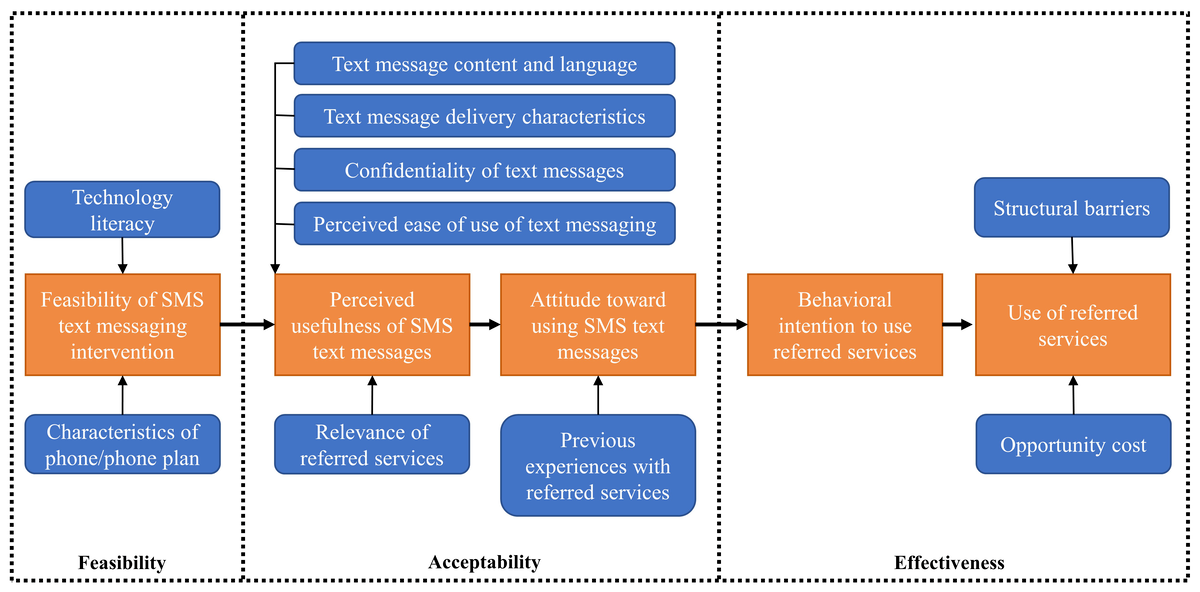
Conceptual model for the SMS text message–based intervention to facilitate patients’ connection to referred services, adapted from the technology acceptance model for resource-limited settings (TAM-RLS). The model connects several patient and intervention inputs (blue boxes) to outcomes (orange boxes) of perceived usefulness of and attitude toward the intervention, as well as intention to use and use of referred services.

This pilot study is guided by the technology acceptance model (TAM), a widely used conceptual framework for modeling user acceptance of technology or technology-based interventions (Figure 1) [42,43]. Owing to unique factors that influence the uptake of technology in underresourced settings, this study further adapted the TAM for resource-limited settings (TAM-RLS) [44]. TAM-RLS posits that perceived usefulness and attitudes toward the use of the SMS text message–based intervention influence the intention to use and actual use of the referred services. The model provides a theoretical scaffolding for the primary outcomes of interest (acceptability and feasibility) and secondary outcome of interest (effectiveness).

Feasibility represents the technical and implementation considerations in executing the intervention [45]. Acceptability consists of patient perspectives on the content, characteristics, and delivery of the SMS text messages [45]. Effectiveness corresponds to the latter 2 outcomes (ie, intention to use and actual use). A patient’s attempt to connect with a referred service represents an intention to use and successful connection represents actual use of that referred service. This study operationalizes the components of feasibility, acceptability, and effectiveness in this model through survey questions, qualitative interview data, and quantitative measures of patient enrollment and referral service use, which together assess all model constructs (Multimedia Appendix 1).

## Methods

### Setting and Community Needs Assessment

This pilot prospective cohort study will be conducted at a FQHC—a publicly funded, community-based health care center for underserved areas—in the southeastern United States, which services a mostly metropolitan, low-income patient population. In 2020, the partnering FQHC saw about 35,000 unique patients, of whom 51% were uninsured and 92% self-identified in electronic health records as racial or ethnic minorities, most of whom identified as Black or Hispanic/Latino. Of the approximately 25,000 patients with known income status, 97% earned incomes at or below 200% of the federal poverty level [46].

Since 2017, case managers with social work licensure have screened and referred patients for unmet social needs using the Protocol of Responding to and Assessing Patients’ Assets, Risks, and Experiences (PRAPARE) within pediatric, adult medicine, and family medicine clinics [47]. One of the most prevalent assessment tools in the United States [48], PRAPARE consists of a validated set of national core measures integrated into the electronic health record, which include patient demographics (eg, race and education) and social needs (eg, housing stability and material security) [49]. Based on identified needs, case managers can refer patients to one or more community-based organizations, government agencies, or services internal to the health center. Since 2019, trained student volunteers have served as community resource navigators to follow up and provide further assistance to patients who received referrals [50]. These volunteers call patients 2 and 4 weeks after the initial referral is made to offer navigation support, as well as record patient’s ability to connect with referred resources.

These efforts to advance screening, referral, and navigation for social needs at the FQHC are the product of a community-academic partnership between the FQHC and a nearby university. At least once a month, providers, navigators, and administrative leadership of the FQHC meet with university researchers to advance initiatives that identify and address needs in the local community.

During these meetings, frontline clinical stakeholders relayed patient requests for the incorporation of SMS into the existing social needs program. The potential benefits of an SMS text message–based intervention were further bolstered by studies of patient-reported barriers [12] and low proportions of resource connection (33%) [10] at the FQHC. With stakeholder support, a convenience sample of 16 patients was surveyed about SMS text messaging during navigator follow-up calls: 87.5% of patients stated that they were comfortable with receiving SMS text messages about referred social needs resources, and 56.3% of them even preferred to receive this information via SMS text message rather than by telephone or in person. Researchers also conducted semistructured interviews with a separate purposive sample of 10 patients, most of whom were amenable to receiving SMS text messages as part of their social care but emphasized that messages should not displace person-to-person relationships with social workers and community resource navigators [51]. This preliminary needs assessment motivated the development of the present SMS text message–based intervention and pilot protocol, which are shaped by regular and ongoing stakeholder engagement.

### Eligibility and Recruitment

Prospective participants will be identified by a case manager during primary care or behavioral health visits at the partnering FQHC. To be eligible, participants must be at least 18 years of age, have at least one referral from a community resource navigator, understand English, have access to a device with an active service plan to send and receive SMS text messages, and be able to provide informed consent. In addition, patients who have already connected with a referred resource prior to enrolling will not be eligible for the study. We will recruit 15-20 patients using a purposive sampling strategy to ensure diversity with regard to age, race, ethnicity, and education, as well as social need characteristics (eg, food and medication assistance). After recruiting 10 patients, the demographic diversity of the sample will be assessed relative to the FQHC’s general primary care population (eg, a majority of Black or Hispanic individuals and food and health care access as leading social needs referrals) [10,46].

Recruitment progress is reported to an advisory committee comprising FQHC clinical leadership, who will determine adjustments to the recruitment protocol to ensure representative participant selection. As participants complete the study, the study team will also engage key clinical stakeholders and FQHC leadership during monthly meetings for interim assessment of the intervention and overall study, discussing participant feedback and further adaptations if necessary. Participants will receive a one-time US $25 gift card after completing the survey and interview for the study.

The primary purpose of this pilot is to determine feasibility and acceptability to refine the use of SMS text message–based outreach in clinical practice and to develop a larger trial, and not to detect significant effects of the intervention on outcomes. A sample size of 15-20 patients was determined to be appropriate for this pilot to allow for recruitment of a sample representative of the broader patient population within a reasonable time frame, given staffing and resource capacity.

### Intervention Overview

**Figure 2:**
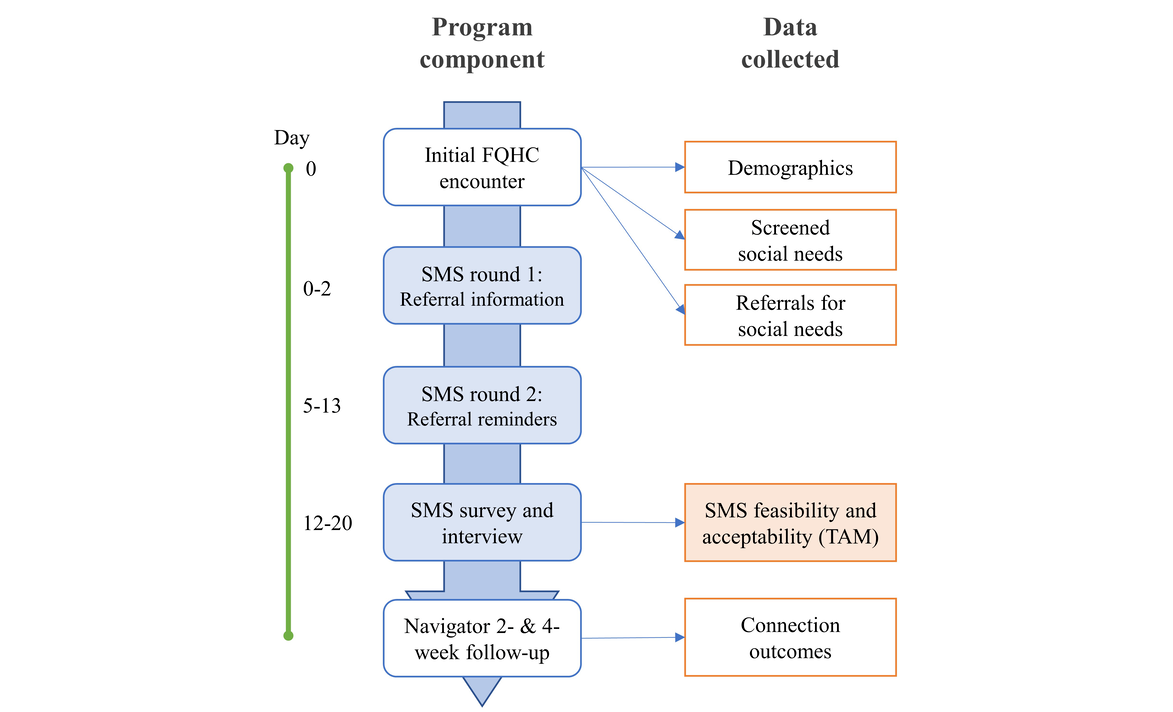
Outline of the SMS text message–based intervention and data collection. Shaded boxes correspond to SMS text message–based intervention components, and unshaded boxes correspond to usual care. FQHC: federally qualified health center; TAM: technology acceptance model.

The goal of the SMS text message–based intervention is to increase the number of patients who attempt to connect with a referred resource in the 2 weeks after referral and, ultimately, increase the number of patients who successfully connect (Figure 2). An intervention mapping approach informed intervention development, incorporating the following: needs assessment, objective and theory selection, message and program design, and pretesting with plans for evaluation and revision [15,52]. In response to preliminary patient input, a key priority in intervention design was ensuring that SMS messages would integrate within existing screening, referral, and navigation activities without the disruption of critical person-to-person interactions and care. Two rounds of SMS text messages will be sent to the enrolled participants. The first round consists of 5-10 messages (depending on the number of referrals) with information about the participant’s referred services for identified unmet social needs, sent upon participant consent within 2 days of screening. The second round of SMS text messages, sent approximately 1 week later to all participants, consists of 2 messages that offer a brief reminder to connect with these services if they have not already. Following the second round of messages, participants will be contacted by a community resource navigator by telephone for follow-up, resuming standard navigation protocol.

SMS text messages will be delivered through Twilio Inc, a third-party web service, integrated into a Research Electronic Data Capture (REDCap) tool database [53,54]. REDCap is a secure, web-based software platform designed to support data capture for research studies. This delivery method was chosen for its relative simplicity, ease of use, and cost-effectiveness at US $0.0075 per message. Many community resource navigation programs already use REDCap to coordinate patient follow-up, including the program at the partnering FQHC [50]. Researchers and clinical staff can schedule automated message triggers (using “Alerts & Notifications”) directly from the database platform without significant technical knowledge or training, making this a suitable and practical approach for future scale-up.

### Message Development and Delivery

Existing community resource navigator scripts informed the design of the message content for both rounds of messages (Table 1). The first round of messages, which will be sent on the day of participant consent, delivers information tailored to their referred resources, including the name, a brief description of the organization or program, and the contact information (eg, telephone numbers, addresses, and URLs) necessary to connect. The second round of messages, which will be sent on Monday morning (5-11 days later or 2-6 business days), offers a brief reminder to attempt to connect with referred resources. This timing is based on clinician and stakeholder input from the FQHC and provides the participant an opportunity to attempt to contact a resource during regular business hours. Message content and referred resource descriptions are automatically populated on the basis of patient data, and messages are automatically sent on the basis of prescheduled triggers (which can be cancelled at any time if the participant chooses to leave the study).

**Table 1:** SMS text message content and timing.

Message category			Message
**Round 1: Information (timing: day of consent, within 2 days of screening)**			
	Message 1: Introduction	Hi [name]! Thank you for signing up. Our team at [FQHC] hopes that these texts can help remind you to reach out to resources that you’ve been referred to.	
	Message 2: Disclaimer and Support	These texts are automated. If you ever want to speak to a person who can help you, please leave a message with [FQHC] Behavioral Health at (000-000-0000).	
	Message 3: Call-back to Conversation	You and [responsible_case_manager] discussed these resource(s) that may be useful for you:	
	Message(s) 4A-4D: Service Descriptions	*Descriptions for 1-4 services that the patient was referred to.* *Example:* SNAP: Federal food program. Apply online at [URL] or by calling [organization] at (000-000-000).	
	Message 5: Conclusion	We hope that you’ll reach out to the resources that you two discussed, if you haven’t already! We’ll send a reminder next week in case it slips your mind.	
**Round 2: Reminders** **(timing: 5-11 days after consent, Monday at 9 AM)**			
	Message 1: Connection Reminder	Good morning! This is a reminder from your care team at [FQHC]. Try to follow up on [provider]’s resources if you haven't already been in touch!	
	Message 2: Data Collection Reminder	You will receive a call soon to learn about your experience with these messages. Thank you for your help and participation!	

### Measures

This study adapts the acceptability of intervention measures to assess attitudes toward SMS text messages [55]. Otherwise, in the absence of validated tools to assess many constructs of technology acceptance for mobile health applications, this study leverages the TAM-RLS to guide the development of measures and facilitate an exploration of feasibility and acceptability tailored to our study and intervention context, the predominant approach in the literature [56].

All data for a given participant will be collected and stored within a secure REDCap database. The participants’ demographic information will be obtained retrospectively from case managers, which includes sex, race, ethnicity, and education. Screening and referral information will also be collected by the case manager, using the PRAPARE screening assessment. Screened social needs include the following domains: food, clothing, phone access, childcare, utilities, medicine or health care access, housing, transportation, employment, stress, and social isolation. Referred services were organized into groups corresponding to the social need domains: food, transportation, housing, financial assistance, utilities, health care access, social or emotional health, childcare, clothing, or phone or technology assistance. For this pilot, referrals to domestic or interpersonal violence organizations are omitted from SMS text messages to protect participant safety.

Three days to a week after the second round of messages, a trained study team member will call the participant to conduct a quantitative survey about their experience with the SMS text messages, including Likert scale measures of attitudes toward message frequency, content, and usefulness (Multimedia Appendix 2). During the call, the team member will also conduct a semistructured qualitative interview to supplement and add depth to the quantitative survey measures (Multimedia Appendix 3). Questions for both the survey and interview are designed to map onto all of the constructs of this study’s conceptual model (Multimedia Appendix 1). The total survey and interview are expected to last 30-45 minutes, and deidentified, encrypted recordings of the qualitative interview and interview notes will be stored securely.

One week after the second round of SMS text messages, the participant will also resume standard of care: a 2- and 4-week follow-up telephonic encounter with a community resource navigator. During these follow-up calls, data about resource connection will be collected as preliminary evidence of effectiveness—this pilot’s secondary outcome.

### Quantitative Analysis

Quantitative survey data about feasibility and acceptability, as well as patient demographic, social needs, and referral characteristics, will be summarized through univariate, descriptive statistics. To determine preliminary effectiveness, a one-sample binomial test will be used to assess whether the proportion of patients who had attempted to contact a referred resource within 2 weeks of the initial referral is greater than 0.57, the corresponding proportion for the FQHC’s screened patients before the pilot.

### Qualitative Analysis

In order to rapidly develop rigorous and actionable evidence for this intervention, qualitative interview data will be analyzed using a rapid qualitative approach, which will employ a thematic analysis informed a priori by the constructs of this study’s conceptual model [57]. Routine discussions and interview debriefs among both the study team and the advising stakeholders will identify areas where further data should be gathered as well as inform the selection of participants. Recordings and interview notes will be coded by 2 trained researchers and reconciled by a third trained researcher to identify themes. As needed, the larger study team will be queried to reconcile differences in interpretation of themes.

### Ethical Considerations

This study received initial approval by the university health system’s institutional review board in May 2021 and an amendment approval in November 2021 (Pro00107167).

## Results

Recruitment of the pilot study began in January 2022. Data collection and analysis will be carried out through the summer of 2022.

## Discussion

### Protocol Overview

We describe a protocol to assess the feasibility and acceptability of an SMS text message–based intervention that integrates automated SMS messages into an existing social needs screening and referral program at an FQHC. This study represents a novel application of an evidence-based intervention medium to promote patients’ connection to referred services to address their identified unmet social needs. This intervention has the potential to provide a valuable touch point for patients seeking to navigate a complex and often fragmented system of health and social services. For patients, SMS text messaging may be a convenient, affordable, and accessible modality to deliver information and reminders about referred services, improving upon solely verbal dissemination methods that create patient-reported approachability barriers (eg, forgetting about resources and lacking the necessary information to connect) [12]. If feasible and acceptable to patients, SMS text messaging may also offer a scalable means of increasing contact between patients and their care teams without overextending organizational capacity, allowing providers to direct their case management efforts to those cases where more intensive approaches would be most valuable.

This pilot has been designed to anticipate and assess a wide range of possible barriers to the implementation of this SMS intervention, as depicted in this study’s TAM-RLS conceptual model. Limited access to or familiarity with SMS within this study’s population, whether a result of financial constraints or restricted opportunities to develop digital literacy, may hamper the feasibility of the intervention at the outset. Issues with SMS text message content, timing, confidentiality, or relative advantage or ease of use may affect participant acceptability, which also depends on the perceived or actual usefulness of the referred services themselves. Larger organizational (eg, staffing, capacity, and practice patterns) and community forces (eg, resource availability, structural barriers, and local policy) also govern whether and how participants are able to connect to referred services, influencing the effectiveness of the intervention. By drawing from the conceptual model, the survey instrument and interview guide will be able to elicit a comprehensive assessment of the factors that interact with this SMS text message–based intervention, which should guide future development and scale. This study’s multi-methods approach also enables a nuanced understanding of patient perspectives through quantitative and qualitative methods, providing practical insights into the design, feasibility, and acceptability of SMS text message–based interventions to improve social and medical care coordination.

This study benefits from a long-standing community-academic partnership that has guided the development of this study and intervention from inception to execution, prioritizing the on-the-ground values and context of the study’s FQHC. Further, this study can build evidence for larger, more overarching initiatives to consolidate care coordination efforts across the sociomedical care continuum. For example, the state of North Carolina—where this pilot will take place—has launched NCCARE360, a digital platform where health care providers and community-based organizations can manage incoming and outgoing referrals, track outcomes, and communicate with enrollees, with platform support for SMS text messaging [58]. SMS text messaging can complement these digital platforms by providing another contact point between referred services and patients, who may otherwise be left out of provider-to-provider communications that occur on the platforms. Scale-up evidence in subsequent studies about the facilitators, challenges, and best practices of automated SMS text messaging may yield insights to guide implementation of these platforms in specific organizations and health centers and facilitate patients’ connection to services organized on the platform.

### Limitations

This study has several limitations. First, the sample size and study design limit the ability to draw conclusions on the effectiveness of the SMS text message–based intervention to promote uptake or initiation of services. The aim of this pilot study is to gather evidence about the feasibility and acceptability of the intervention, and the relative effectiveness of the intervention will instead be the focus of a later scale-up trial. Second, the external validity of these findings may be limited by the single-site study design, and the results of the pilot should be expanded by investigation of similar interventions in different settings, program models, and patient populations. In particular, electronic referral platforms—not currently used by this study’s partnering FQHC—represent a prominent and advancing innovation where automated messaging and platform-embedded connection outcomes should be studied. Third, more specific measures related to internet accessibility, literacy, privacy, and use—as well as reasons for nonparticipation in general—should be explored to understand broader issues in digital inclusion, especially as smartphones become an increasingly prominent and expected part of everyday life. At the moment, many screening tools including PRAPARE only collect limited information about technological and broadband access. Finally, this intervention uses unidirectional as opposed to bidirectional SMS text messages. Though previous literature has suggested the effectiveness of bidirectional SMS text messages [59], bidirectional messaging posed additional technical and workforce capacity demands.

### Conclusions

This intervention aims to explore SMS text messaging as a new potential opportunity for an accessible, affordable, and scalable approach to improving care for patients with unmet social needs, contributing to an expanding evidence base about best practices for incorporating social needs screening and response interventions in clinical settings [60]. The detailed methods described in this protocol may be of interest for researchers and practitioners seeking to incorporate automated SMS text messaging within an existing social needs screening and referral program, with the goal of augmenting rather than disrupting or displacing interpersonal care for an underresourced patient population.

## Appendix

Multimedia Appendix 1 Model Constructs and Associated Measures.

Multimedia Appendix 2 Quantitative Exit Survey for SMS Pilot.

Multimedia Appendix 3 Qualitative Interview Guide for SMS Pilot.

## Abbreviations

FQHC: federally qualified health center
PRAPARE: Protocol of Responding to and Assessing Patients’ Assets, Risks, and Experiences
REDCap: Research Electronic Data Capture
TAM: technology acceptance model
TAM-RLS: technology acceptance model for resource-limited settings

## Data Availability

The data set generated during the current study is not publicly available owing to the institutional review board’s restrictions for personal health information, but a limited, deidentified data set is available from the corresponding author on reasonable request.
