# Supplementary material for: A Tailored SMS Text Message–Based Intervention to Facilitate Patient Access to Referred Community-Based Social Needs Resources: Protocol for a Pilot Feasibility and Acceptability Study
Source: JMIR Res Protoc. 2022 Oct 11;11(10):e37316. doi: 10.2196/37316 (PMC9597426; doi:10.2196/37316)
Supplement: Multimedia Appendix 1 [file resprot_v11i10e37316_app1.docx]

**Supplementary Material 1 for SMS Pilot Protocol**

Table S1. Model Constructs and Associated Measures

| **Construct** | **Definition** | **Measures** |
| --- | --- | --- |
| **Feasibility** | | |
| **Implementation Feasibility of SMS Intervention** | Extent to which the SMS-based intervention can be practically implemented as proposed. | Proportion of eligible patients enrolled over all eligible enrollment attempts and reasons for ineligibility. |
|  |  | At the start of the program, we sent text messages with information about your referrals on [date]. Do you remember receiving these text messages? |
|  |  | About a week later, on [date], we sent a second set of text messages with a reminder about your referrals. Do you remember receiving these text messages? |
| Characteristics of Phone and Phone Plan | Features of device hardware or service plan, including type of phone, and any challenges due to device damage or service lapses. | What device did you use to receive text messages for this program? |
|  |  | Between the start of the program and now, did you experience any challenges or changes with your phone (e.g., lost, broken) that prevented you from receiving or reading text messages? |
|  |  | Between the start of the program and now, did you experience any challenges or changes with your phone number or phone plan (e.g., changed number, ran out of minutes) that prevented you from receiving or reading text messages? |
| Technology Literacy | Familiarity with SMS messaging technology prior to the initiation of this study. | Before the start of this program, how often did you send or receive text messages? |
|  |  | Before the start of this program, how comfortable were you with sending or receiving text messages? |
| **Acceptability** | | |
| **Perceived Usefulness of SMS Messages** | Perception of overall utility of SMS messages for receiving information and reminders about referred services. | How useful were the text messages for providing you with information about [resource name]? |
|  |  | How useful were the text messages for reminding you to call, visit, or contact [resource name]? |
|  |  | Did the messages have the information that you needed to reach out to [resource name]? |
|  |  | I shared the information in the text messages with others. (SD-SA) |
| **Attitude Towards Using (Opting Into) SMS Messages [Weiner, 2017]** | Overall feelings, opinions, and beliefs about receiving SMS messages with information and reminders about referred services. | I liked receiving these text messages about resource(s) from the navigator. (SD-SA) |
|  |  | The process of receiving these text messages met my approval. (SD-SA) |
|  |  | Receiving text messages about resource(s) from my navigator is appealing to me. (SD-SA) |
|  |  | I welcome the use of text messages like these as part of my care. (SD-SA) |
|  |  | Text messaging is a good way for me to receive information about resources from my case manager. (SD-SA) |
|  |  | Text messaging is a good way for me to receive reminders about resources from my case manager. (SD-SA) |
|  |  | *Based on your experience, do you think text messages about resources like [resource names] are something all patients should receive? Why? Why not?* |
| SMS Message Content and Language | Substance and presentation of information within SMS messages, including comprehensibility and phrasing. | It was easy to read and understand these text messages. (SD-SA) |
| SMS Delivery Characteristics | Features of SMS message delivery, including frequency, quantity, and timing. | *We sent two rounds of texts, one week apart from one another. How often would you have wanted to receive texts from this program?* |
|  |  | *We sent texts over a period of two weeks after your conversation with your case*  *manager. Would it have been helpful to receive texts for a longer or shorter period of time?* |
|  |  | *What would be the best number of texts to receive from this program?* |
|  |  | *We sent the second round of texts at 9AM on Tuesday morning. How did you feel about the timing of these texts? What would be the best time and day of the week to receive these texts?* |
|  |  | When would you read these text messages? |
| Confidentiality of SMS Messages | Perceptions or concerns related to privacy of information received in SMS messages. | I had concerns about the privacy of my information sent over text message. (SD-SA) |
| Perceived Ease of Use of SMS Messages | Perception of difficulties or lack thereof in the process of receiving information about referred services via SMS. | I prefer to be texted information about these resources, as opposed to being called about the same information. (SD-SA) |
|  |  | I prefer to be texted reminders to reach out to these resources, as opposed to being called about the same reminders. (SD-SA) |
|  |  | *In what situations would you like your care team at [clinic] to send you a text message about these resources, and in what situations would you prefer a phone call or an in-person appointment?* |
| Relevance of Referred Services | Perceived pertinence of referred services in relation to participants’ identified needs. | I thought the resources described in the text messages were relevant to me. (SD-SA) |
| Previous Experiences with Referred Services | Any past attempts the participant has made to obtain services they perceive as similar to those offered in the referral and the result of those attempts. | *Have you ever attempted before to get support similar to the referrals you received texts about? How did that experience affect your ability to seek services like it in the future?* |
| **Effectiveness** | | |
| **Behavioral Intention to Use Referred Services** | Demonstrated intention to connect with and initiate referred services, described in the SMS messages. | Have you tried to call, visit, or otherwise make contact with [resource name]? |
|  |  | When you did you first try to call or visit? |
|  |  | Proportion of participants who attempted to connect with at least one referred service, compared to a non-intervention baseline. |
| **Actual Use of Referred Services** | Initiation of referred services for identified unmet social needs. | *Please describe how you found the text messages helpful in connecting with the resources you and the navigator discussed together.* |
|  |  | *Please describe how you found the text messages unhelpful in connecting with these resources. What challenges did you face?* |
|  |  | Proportion of participants who successfully connected with at least one referred service, compared to a non-intervention baseline. |
| Structural Barriers | System- or organization-level obstacles that impede or delay the initiation of services. | Was there a reason you could not call or visit [resource name]? |
| Opportunity Cost | Perceived trade-offs to accessing referred services, including time, finances, convenience, and other competing priorities. | Was there a reason you could not call or visit [resource name]? |

(SD-SA) = Respondents asked to evaluate the given statement on a 5-point scale from “strongly disagree” to “strongly agree.” Constructs in bold text are outcomes. Measures in italic text are questions included in the qualitative semi-structured interview; the remaining measures are quantitative survey questions or calculated proportions.
