# Supplementary material for: A Tailored SMS Text Message–Based Intervention to Facilitate Patient Access to Referred Community-Based Social Needs Resources: Protocol for a Pilot Feasibility and Acceptability Study
Source: JMIR Res Protoc. 2022 Oct 11;11(10):e37316. doi: 10.2196/37316 (PMC9597426; doi:10.2196/37316)
Supplement: Multimedia Appendix 2 [file resprot_v11i10e37316_app2.docx]

**Quantitative Exit Survey for SMS Pilot**

***To be emailed to patients for completion.***

**Fidelity and Feasibility**

1. Before the start of this program, how often did you send or receive text messages?
   1. Every day
   2. A few times a week
   3. A few times a month
   4. Less than once a month
   5. Never
   6. Unsure
2. Before the start of this program, how comfortable were you with sending or receiving text messages? Please select the statement that best applies to you.
   1. Not comfortable at all
   2. Not comfortable
   3. Neutral
   4. Comfortable
   5. Very comfortable
   6. Unsure
3. At the start of the program, we sent text messages with information about your referrals on [date_cons]. Do you remember receiving these text messages?
   1. Yes (1)
   2. No (0)
   3. Unsure (99)
4. About a week later, on [date_sms2], we sent a second set of text messages with a reminder about your referrals. Do you remember receiving these text messages?
   1. Yes
   2. No
   3. Unsure
5. [If “Yes” to Q3 or Q4]: When would you read these text messages?
   1. As soon as you saw them
   2. Later that day
   3. Later that week
   4. More than one week later
   5. Never
   6. Unsure
6. What device did you use to receive text messages for this program?
   1. Basic cell phone (non-smartphone)
   2. Smartphone
   3. Tablet
   4. Program on computer or laptop
   5. Other: (please specify)
7. Between the start of the program and now, did you experience any challenges or changes with your phone (e.g., lost, broken) that prevented you from receiving or reading text messages?
   1. Yes
   2. No
   3. Unsure
8. Between the start of the program and now, did you experience any challenges or changes with your phone number or phone plan (e.g., changed number, ran out of minutes) that prevented you from receiving or reading text messages?
   1. Yes
   2. No
   3. Unsure

**Attitudes and Message Content**

Please choose the response that best describes your perspective and experience with this text messaging program.

1. I liked receiving these text messages about resource(s) from the navigator.
2. The process of receiving these text messages met my approval.
3. Receiving text messages about resource(s) from my navigator is appealing to me.
4. I welcome the use of text messages like these as part of my care.
5. It was easy to read and understand these text messages.
6. I thought the resources described in the text messages were relevant to me.
7. I had concerns about the privacy of my information sent over text message.
8. I shared the information in the text messages with others.
   1. Strongly disagree
   2. Disagree
   3. Neither agree nor disagree
   4. Agree
   5. Strongly agree
   6. Not applicable

**Usefulness and Effectiveness** [Repeats for each referred resource.]

Please answer the following questions about [resource name,] which you and the navigator thought may be helpful for you.

1. How useful were the text messages for providing you with information about [resource name]?
   1. Not useful at all
   2. Barely useful
   3. Somewhat useful
   4. Very useful
   5. Unsure
2. How useful were the text messages for reminding you to call, visit, or contact [resource name]?
   1. Not useful at all
   2. Barely useful
   3. Somewhat useful
   4. Very useful
   5. Unsure
3. Did the messages have the information that you needed to reach out to [resource name]?
   1. Yes
   2. No
   3. Unsure
4. Have you tried to call, visit, or otherwise make contact with [resource name]?
   1. Yes
   2. No
   3. Unsure
5. [If yes:] When you did you first try to call or visit?
   1. After the call with the navigator.
   2. After first round of texts
   3. After second round of texts
   4. Unsure
6. [If no:] Was there a reason you could not call or visit? (Select all that apply)
   1. I forgot.
   2. I am no longer interested or need the service.
   3. I have not had time yet.
   4. I had trouble with my phone or phone plan.
   5. I had trouble with accessing the internet.
   6. I had trouble with transportation.
   7. No specific reason.
   8. Other: (please elaborate)

**Attitudes and Platform**

Please choose the response that best describes your perspective and experience with this text messaging program.

1. Text messaging is a good way for me to receive information about resources.
2. Text messaging is a good way for me to receive reminders about resources.
3. I prefer to be texted information about these resources, as opposed to being called about the same information.
4. I prefer to be texted reminders to reach out to these resources, as opposed to being called about the same reminders.
   1. Strongly disagree
   2. Disagree
   3. Neither agree nor disagree
   4. Agree
   5. Strongly Agree
   6. Not applicable
5. In the next week, when would be a good time to follow up by phone, for a thirty minute conversation? After completing this survey and the call, you will be rewarded your $25 gift card.
