# Supplementary material for: A Tailored SMS Text Message–Based Intervention to Facilitate Patient Access to Referred Community-Based Social Needs Resources: Protocol for a Pilot Feasibility and Acceptability Study
Source: JMIR Res Protoc. 2022 Oct 11;11(10):e37316. doi: 10.2196/37316 (PMC9597426; doi:10.2196/37316)
Supplement: Multimedia Appendix 3 [file resprot_v11i10e37316_app3.docx]

**Qualitative Interview Guide for SMS Pilot**

***To be administered over the phone.***

**Introduction**

- “Hello! This is [insert name] calling from Duke University. May I please speak to [insert name]?”
  - If it is the person: “Hi there! Could you confirm your DOB for me?”
  - “Great! I’m calling to get updates on how the texting platform has been for you! May I take about 30 minutes of your time to discuss that so we can understand your experience receiving our texts?
  - If yes: “Great! How has your experience with the text messages been overall?”
  - If no: “When would be a better time for you?”

**Usefulness and Effectiveness**

1. Please describe how you found the text messages helpful in connecting with the resources you and the navigator discussed together.
2. Please describe how you found the text messages unhelpful in connecting with these resources. What challenges did you face?

**Acceptability and Platform**

1. Right now, we are trying to experiment with different ways of communicating information and reminders about resources like [...] to patients. In what situations would you like your care team at Lincoln to send you a text message about these resources, and in what situations would you prefer a phone call or an in-person appointment?
   1. Probe for: relevance of convenience, cost-effectiveness, ease of use. Is running out of minutes on your phone’s voice plan an important concern?
   2. Probe for: relevance of privacy, comprehensibility, and language and tone, as indicated by their survey responses.
   3. Probe for: human-to-human interaction.
2. Based on your experience, do you think text messages for resources like [...] are something all patients should receive? Why? Why not?”

**Timing and Frequency**

1. We sent two rounds of texts, one week apart from one another. How often would you have wanted to receive texts from this program? Every day? Every two weeks?
2. We sent texts over a period of two weeks after your conversation with the navigator. Would it have been helpful to receive texts for a longer period of time (e.g., a month)? Shorter?
3. What would be the best number of texts to receive from this program?
4. We sent the second round of texts at 9AM on Monday morning. How did you feel about the timing of these texts? What would be the best time to receive these texts? What would be the best day of the week?

**Final Recap**

1. Have you ever attempted before to get support similar to the referred services you received texts about? How did that experience affect your ability to seek services like it in the future?
2. What improvements would you suggest for the whole process in general, from getting referred to services by the Lincoln navigator to now?
3. Is there anything else that you would like the care team at Lincoln to know about the text messages?

**Reward Logistics**

1. “Thanks again for participating in the study! As promised, you’ll be receiving a [describe gift card].”
